# Anti-tumoral activity of single and combined regorafenib treatments in preclinical models of liver and gastrointestinal cancers

**DOI:** 10.1038/s12276-019-0308-1

**Published:** 2019-09-24

**Authors:** Flavia Fondevila, Carolina Méndez-Blanco, Paula Fernández-Palanca, Javier González-Gallego, José L. Mauriz

**Affiliations:** 1 Institute of Biomedicine, University of León, León, Spain; 2 Centro de Investigación Biomédica en Red de Enfermedades Hepáticas y Digestivas (CIBERehd), Madrid, Spain

**Keywords:** Liver cancer, Targeted therapies, Colorectal cancer, Cancer models

## Abstract

Regorafenib is a sorafenib-derived chemotherapy drug belonging to the multikinase inhibitor family. This agent effectively targets a wide range of tyrosine kinases involved in cancer biology, such as those implicated in oncogenesis, angiogenesis, and tumor microenvironment control. The beneficial effects of regorafenib in clinical trials of patients who suffer from advanced hepatocellular carcinoma (HCC), colorectal cancer (CRC) or gastrointestinal stromal tumors (GISTs) refractory to standard treatments led to regorafenib monotherapy approval as a second-line treatment for advanced HCC and as a third-line treatment for advanced CRC and GISTs. Multiple in vitro and in vivo studies have been performed over the last decade to reveal the molecular mechanisms of the favorable actions exerted by regorafenib in patients. Given the hypothetical loss of sensitivity to regorafenib in tumor cells, preclinical research is also searching for novel therapeutic approaches consisting of co-administration of this drug plus other agents as a strategy to improve regorafenib effectiveness. This review summarizes the anti-tumor effects of regorafenib in single or combined treatment in preclinical models of HCC, CRC and GISTs and discusses both the global and molecular effects that account for its anti-cancer properties in the clinical setting.

## Regorafenib: Combination therapy in liver and gastrointestinal cancer

The cancer drug regorafenib exhibits a broad range of anti-tumor activities that could be enhanced by combination with other treatments. A team led by José L. Mauriz from the University of León, Spain, review the ways in which regorafenib, blocking several enzymes involved in cancer biology, has been shown to shrink tumors in different models of liver, colon and gastrointestinal cancer. Its mechanisms of action include blockade of new blood vessel formation, induction of cell death and modulation of the immune microenvironment. Research studies show that co-administration of regorafenib with other drugs directed at various molecular targets or immune pathways produces synergistic effects against cancer cells. The preclinical data highlights the potential of combination drug regimens to improve outcomes among patients eligible for regorafenib treatment.

## Introduction

Regorafenib is an orally available multitargeted tyrosine kinase inhibitor (TKI)^1–3^ that emerged from the process of optimizing sorafenib efficacy by modulating its molecular structure^4^. The difference between these TKIs is the addition of a unique fluorine atom to the central phenyl ring of sorafenib^4^, which confers a broader inhibitory profile and greater pharmacological activity to regorafenib^5^. Regarding sorafenib and regorafenib common targeting profile, both drugs share the ability to inhibit tyrosine kinases involved in signaling pathways driving tumorigenesis and cancer progression. These kinases are isoforms of rapidly accelerated fibrosarcoma (RAF) RAF-1, B-RAF, vascular endothelial growth factor receptor (VEGFR) 1–3, and platelet-derived growth factor receptor (PDGFR) β^6–8^. However, regorafenib also blocks additional tyrosine kinases indispensable for oncogenesis, angiogenesis and tumor microenvironment maintenance in addition to those previously mentioned. In line with this effect, regorafenib inhibits B-RAFV600E (a mutant isoform of B-RAF), the oncogenic kinases KIT and RET, angiopoietin 1 receptor (TIE2)^2,6,9^, PDGFRα and fibroblast growth factor receptors (FGFRs) 1 and 2^1,4^. It has been proposed that sorafenib may even target B-RAFV600E, KIT and RET kinases^6,7^; nonetheless, inhibition would require much higher sorafenib doses than regorafenib doses^6^. These results were also reported when comparing the capability of both TKIs to suppress the tyrosine kinases included in the common inhibitory profile^6^; thus, sorafenib is an inhibitor with weaker kinase affinity. The blockade of a high number of tyrosine kinases that cooperate in tumor initiation and progression, in addition to the stronger inhibitory effectiveness of this drug than sorafenib, means that regorafenib is a more potent TKI with promising applications in cancer when standard chemotherapies fail. In fact, as a consequence of its high efficacy in clinical trials, regorafenib was approved as second-line therapy for advanced hepatocellular carcinoma (HCC)^4,5,10^ and as a third-line treatment for advanced colorectal cancer (CRC)^4,9,11^ and gastrointestinal stromal tumors (GISTs)^4,12^. Apart from updating the current therapeutic landscape for advanced-stage HCC, CRC, and GISTs, this review focuses on the anti-tumor actions of regorafenib monotherapy in HCC, CRC, and GISTs preclinical models, as well as on the anti-cancer properties resulting from combining this drug with other compounds as new treatment approaches against these cancer types, detailing not only the global effects but also the molecular mechanisms.

## Regorafenib and HCC

### Therapeutic approaches for advanced HCC

**Fig. 1:**
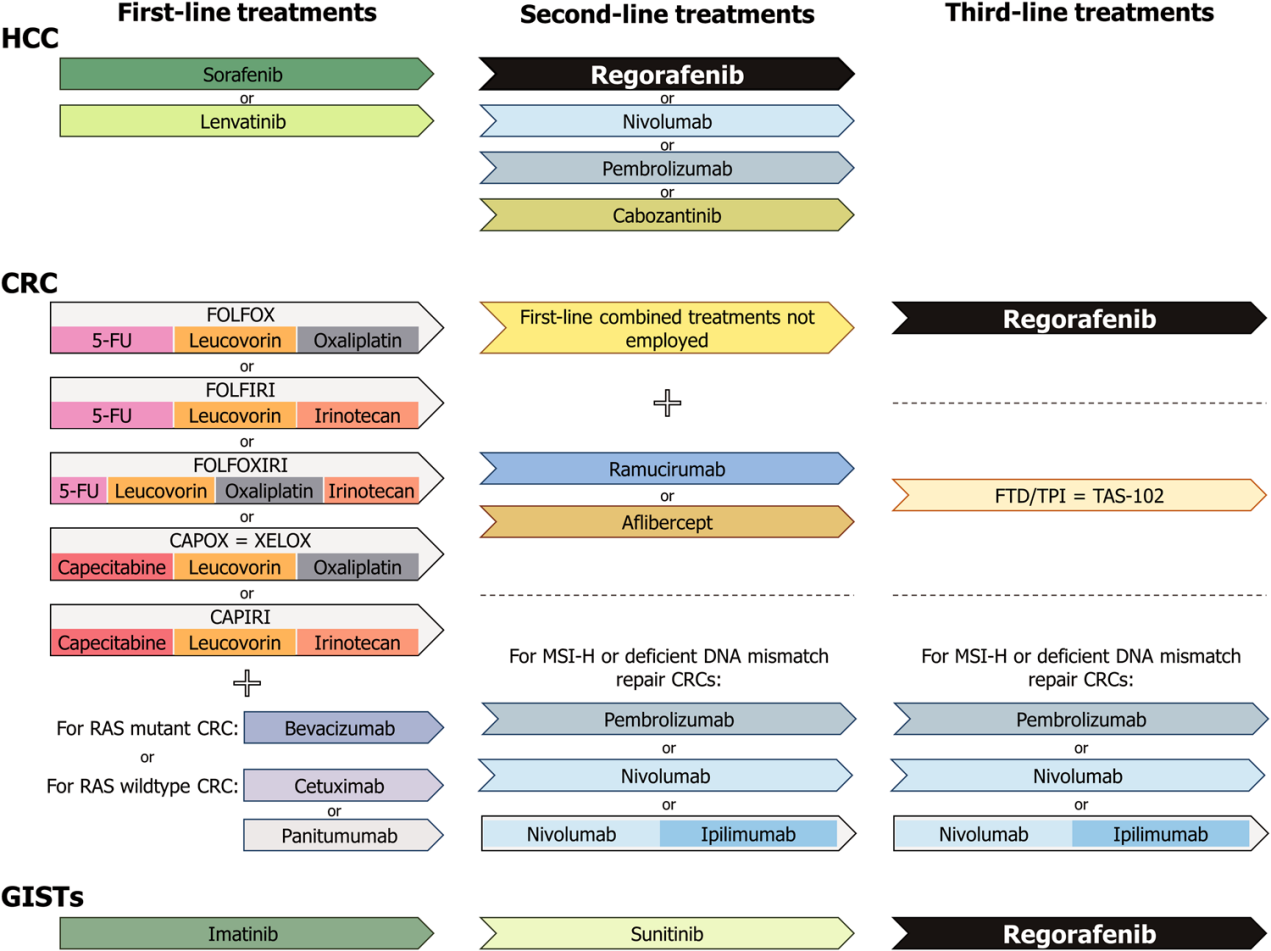
Sequential treatment schedule comprising the currently approved chemotherapeutic options for HCC, CRC, and GISTs in advanced stages. 5-FU: 5-fluorouracil; CRC: colorectal cancer; FTD: trifluridine; GISTs: gastrointestinal stromal tumors; HCC: hepatocellular carcinoma; MSI-H: microsatellite instability-high; TPI: tipiracil

Liver cancer ranks as the sixth most diagnosed cancer and fourth leading cause of cancer-related death worldwide^13^. A total of 75–85% of primary liver tumors are classified as HCC^13^, a major health problem whose incidence and mortality rates are continuously increasing^8,10,14^. The main etiological factors for HCC are hepatitis B or C virus infection, aflatoxin B1 exposure, alcohol intake, and metabolic syndrome related to obesity and type 2 diabetes^5,13^. HCC is usually diagnosed at advanced stages, when no healing treatments are available^8,15^; the survival rate at 5 years is only 3%^8^, and sorafenib remained the only approved front-line treatment until 2018^5^ (Fig. 1). Systemic treatment with sorafenib has become the standard therapy for advanced HCC^16,17^ since the Food and Drug Administration (FDA) approved it in 2007^8^, and this treatment has prolonged overall survival (OS) by almost 3 months^17^. Despite this breakthrough for the management of advanced HCC^5^, sorafenib has important shortcomings, such as its low response rate^16^. This is related to the development of somatic mutations by HCC cells after prolonged treatment and the resulting drug resistance acquisition^15,18^.

There have been multiple attempts to develop molecular targeted drugs better than sorafenib capable of acting as alternatives in the first-line setting or even in the second-line setting after sorafenib failure. However, every phase 3 trial assessing new single-agents from 2007 to 2016 obtained negative results and did not achieve statistical superiority over sorafenib^10,16^. Regarding first-line treatment options apart from sorafenib, lenvatinib was recently approved for patients with advanced HCC, being the unique drug that has demonstrated mild positive outcomes^8,16^ (Fig. 1). Eight second-line placebo-controlled phase 3 clinical trials were conducted in patients who progressed after sorafenib administration, but all of them were ineffective^16^. It was not until the publication of regorafenib results in 2017 and its consecutive FDA approval that the first encouraging outcomes were found in this field^10,16^ (Fig. 1). Regorafenib was evaluated in the randomized, double-blind, placebo-controlled phase 3 RESORCE trial, where this drug demonstrated efficacy and safety in patients with HCC who experienced disease progression during sorafenib treatment. This trial was the first to indicate a significant OS benefit compared with placebo in HCC patients refractory to sorafenib, with an OS of 10.6 months in the regorafenib group versus 7.8 months in the placebo group (HR = 0.63, CI 0.50–0.79, *p* < 0.0001)^19^.

The FDA newly authorized cabozantinib^20^ and granted accelerated but conditional approval of two human IgG4 monoclonal antibodies against programmed cell death-1 (PD-1), nivolumab and pembrolizumab, as second-line treatments for advanced HCC^5,8,17^ (Fig. 1). Additional clinical trials testing different molecular targeted agents and immune checkpoint inhibitors are currently ongoing, and better outcomes are expected to expand the therapy spectrum for HCC^5,8,14,16^.

### Regorafenib for advanced HCC: evidences in preclinical models

The results from RESORCE suggested high pharmacological activity of regorafenib when sorafenib is ineffective^5^. Considering this finding, experimental research has focused on determining the anti-tumor effects of regorafenib on preclinical models of HCC. A preliminary study with Hep3B, HepG2, and PLC/PRF/5 human HCC cell lines revealed that regorafenib reduces cell proliferation in a concentration- and time-dependent manner^21^. It was found that Hep3B and PLC/PRF/5 cells respond similarly to the drug, having an IC_50_ near 5 µM for regorafenib, while HepG2 cells exhibited greater sensitivity, with an approximate IC_50_ value of 1 µM^21,22^. Moreover, 10-fold lower doses of regorafenib than previously mentioned were needed to decrease alpha-fetoprotein (AFP) levels in AFP-positive HepG2 and PLC/PRF/5 cells, which supports the inhibitory activity of regorafenib on HCC cells proliferation^22^. Likewise, Tsai et al.^23^ and Liu et al.^24^ independently demonstrated the dose- and time-dependent cytotoxic effects of this drug in the SK-HEP-1 HCC cell line, whereas Tai et al.^25^ showed similar results in HA59T cells. Regorafenib administration at 20 mg/kg/day resulted in anti-HCC effects in PLC/PRF/5^25^, SK-HEP-1/*luc2*, and Hep3B 2.1-7-bearing subcutaneous xenograft mice, suppressing tumor growth^25,26^ as well as reducing tumor weight and size^25^. Furthermore, regorafenib administered once daily at 10 mg/kg has been shown to be enough to significantly extend survival time in an H129 hepatoma in vivo model and delay tumor growth in 8/10 patient-derived (PD) HCC xenograft (HCC-PDX) mouse models^27^.

It has been reported that regorafenib induces cell death by apoptosis in a concentration-dependent manner in Hep3B^21,25^, PLC/PRF/5, HepG2, SK-HEP-1, and HA59T HCC cells^25^. This agent reduced Bcl-2, Bcl-2-like protein 1 (Bcl-xL), survivin^21^, induced myeloid leukemia cell differentiation protein (Mcl-1), and cyclin D1^25^ protein levels; enhanced caspases 3, 8, and 9 cleavage; and stimulated pro-apoptotic Bcl-2 associated X (Bax) expression^21^ and poly(ADP-ribose) polymerase (PARP) activation^25^. Consistent with these results, the apoptotic mediator phospho-c-Jun N-terminal kinase (JNK) and its target phospho-c-Jun were upregulated in Hep3B cells after regorafenib treatment^21^. Additional data from HCC in vivo studies indicated that apart from downregulation of full-length PARP, regorafenib can increase the expression of the pro-apoptotic Bcl-2-associated agonist of cell death (BAD)^27^ and decrease cyclin D1 levels^26^. On the other hand, this drug enhanced microtubule-associated protein 1 light chain 3 II (LC3-II) and Beclin 1 expression in Hep3B cells, suggesting the induction of autophagy, a process that is related to drug-mediated tumor cell growth inhibition^21^. These results are supported by a study performed with HepG2 and Hep3B cell lines where regorafenib activated pro-death autophagy due to protein kinase B (AKT)/mammalian target of rapamycin (mTOR) signaling abrogation^28^.

The transducer and activator of transcription 3 (STAT3)-related signaling pathway, which includes some of the previously mentioned anti-apoptotic proteins, has been strongly associated with cancer progression and advanced-stage HCC. Regorafenib has been shown to downregulate this pathway by directly relieving the autoinhibited N-Src homology region 2 (SH2) domain of SH2 domain-containing phosphatase 1 (SHP-1), a negative regulator of STAT3, in both in vitro and in vivo HCC models^25^. Constitutive activation of nuclear factor-ĸB (NF-ĸB) in cancer cells, which depends on upstream kinases such as extracellular signal-regulated kinase (ERK), has been linked to downstream anti-apoptotic proteins upregulation and apoptosis evasion. An in vitro investigation using SK-HEP-1 cells reported that regorafenib suppresses the expression of NF-ĸB, anti-apoptotic X-linked inhibitor of apoptosis (XIAP), Mcl-1 and cellular FLICE-like inhibitory protein (c-FLIP) while increasing cytochrome *c* levels and the sub-G1 cell population^23^. Regarding ERK protein levels, regorafenib inhibited ERK phosphorylation in SK-HEP-1^23^, Hep3B^21,22^, and PLC/PRF/5 cells^21^. Indeed, regorafenib was found to reduce the expression of phospho-mitogen-activated protein kinase (MAPK) 7 (MEK), an upstream molecule of ERK^22^. All this evidence seems to indicate that regorafenib induces both extrinsic and intrinsic apoptotic pathways through suppressing ERK/NF-ĸB activation^23^. Such findings agree with those observed in an in vivo study employing SK-HEP-1/*luc2* and Hep3B 2.1-7-bearing mice^26^. In contrast, although most investigations have demonstrated that regorafenib abrogates RAS/RAF/MEK/ERK signaling, there is in vivo evidence showing that the drug can promote the phosphorylation of several components of the MAPK pathway. This unexpected induction, which is accompanied by an uncommon increase in the proliferation marker pS10 histone H3, has to be thoroughly analyzed^27^.

Sustained angiogenesis, invasion and metastasis constitute key cancer hallmarks whose effector proteins are also modulated by NF-ĸB. Regorafenib has been shown to block this transcription factor signal cascade and drive anti-angiogenic and anti-metastatic effects in SK-HEP-1 cell line^24^. The results obtained included decreased levels and secretion of the angiogenesis-associated proteins VEGF, tumor necrosis factor-α (TNF-α), interleukin-1β (IL-1β), and IL-6; reduced expression and secretion of the metastasis-associated markers matrix metalloproteinase-2 (MMP-2) and MMP-9; and inhibition of cell invasion^24^. Furthermore, Chen et al.^29^ disclosed that regorafenib impedes epithelial-to-mesenchymal transition (EMT) through suppressing the ERK and STAT3 pathways and consequent inhibition of hepatocyte growth factor (HGF)-mediated Snail upregulation in HCC cells. These anti-angiogenic and anti-metastatic properties were supported by data from in vitro assays using Hep3B, HepG2, and PLC/PRF/5 cells, as well as by results from an in vivo experiment using SK-HEP-1/*luc2* and Hep3B 2.1-7-bearing mouse models^26^. In addition, regorafenib has been shown to control the balance between MMPs and endogenous tissue inhibitors of metalloproteinases (TIMPs), a critical ratio that determines invasion and metastatic potential, in both SMMC-7721 and Hep3B cell lines^30^.

Regarding the possibility that regorafenib could have some effect on immune-related proteins, this drug has been shown to attenuate the expression of PD-1 ligand 1 (PD-L1) checkpoint in BALB/c nude mice inoculated with Hep3B cells. Downregulation of immunosuppressive molecules such as PD-L1 could lead to reactivation of the immune response against HCC and prevent the characteristic immune evasion of tumor cells^31^. Moreover, Carr et al.^21^ reported that incubation with regorafenib for several weeks causes Hep3B cell quiescence. This finding could supply a useful model to investigate dormancy, a clinical issue that most likely hides behind cancer recurrence after definitive primary treatment in patients^21^.

**Fig. 2:**
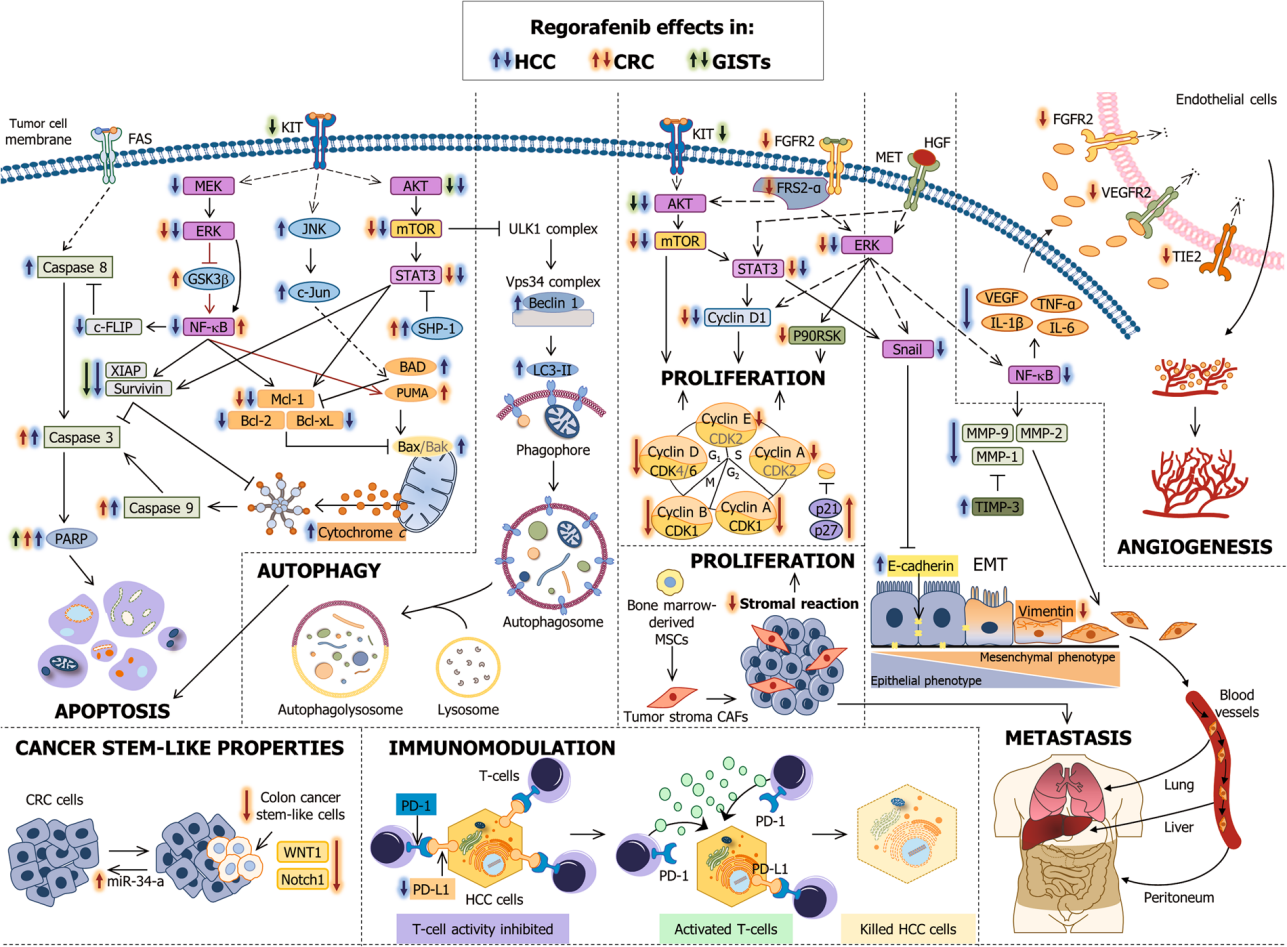
Effect of regorafenib on the main cancer-related signaling pathways and processes involved in HCC, CRC, and GISTs tumor cells survival. AKT: protein kinase B; BAD: Bcl-2 associated agonist of cell death; Bak: Bcl-2 antagonist/killer; Bax: Bcl-2 associated X; Bcl-xL: Bcl-2-like protein 1; c-FLIP: cellular FLICE-like inhibitory protein; CAF: carcinoma-associated fibroblast; CDK1: cyclin-dependent kinase 1; CDK6: cyclin-dependent kinase 6; CRC: colorectal cancer; EMT: epithelial-to-mesenchymal transition; ERK: extracellular signal-regulated kinase; FAS: tumor necrosis factor receptor superfamily member 6; FGFR2: fibroblast growth factor receptor 2; FRS2-α: FGFR substrate 2-α; GISTs: gastrointestinal stromal tumors; GSK3β: glycogen synthase kinase 3β; HCC: hepatocellular carcinoma; HGF: hepatocyte growth factor receptor; IL-1β: interleukin-1β; IL-6β: interleukin-6β; JNK: c-Jun N-terminal kinase; LC3-II: microtubule-associated protein 1 light chain 3 II; Mcl-1: induced myeloid leukemia cell differentiation protein; MEK: MAPK 7; MET: HGF receptor; MMP-1: matrix metalloproteinase-1; MMP-2: matrix metalloproteinase-2; MMP-9: matrix metalloproteinase-9; MSC: mesenchymal stem cell; mTOR: mammalian target of rapamycin; NF-ĸB: nuclear factor-ĸB; Notch1: Notch receptor 1; P90RSK: 90-kDa ribosomal protein S6 kinase 1; PARP: poly(ADP-ribose) polymerase; PD-1: programmed cell death-1; PD-L1: PD-1 ligand 1; PUMA: p53-upregulated modulator of apoptosis; SHP-1: SH2 domain-containing phosphatase 1; STAT3: transducer and activator of transcription 3; TIE2: angiopoietin 1 receptor; TIMP-3: MMP inhibitor-3; TNF-α: tumor necrosis factor-α; ULK-1: unc-51 like autophagy activating kinase 1; VEGF: vascular endothelial growth factor; VEGFR: VEGF receptor; Vps34: PI3K catalytic subunit type 3; WNT1: Wnt family member 1; XIAP: X-linked inhibitor of apoptosis

In light of these results, the inhibition of tumor growth, angiogenesis and metastasis, in addition to cell apoptosis promotion and immune response recovery, explains the great anti-tumoral activity exerted by regorafenib in HCC (Fig. 2). Its considerable ability to target cancer-related processes and signaling pathways highlights the importance of regorafenib administration in cases of sorafenib-insensitive advanced HCC patients.

### Emerging combined treatment strategies with regorafenib against advanced HCC

Although clinical and preclinical data have demonstrated great regorafenib efficacy against HCC, several regorafenib-combined therapies targeting parallel pathways have emerged with the aim of improving its anti-tumor actions and resolving its main drawbacks. Given that overactivation of the phosphatidylinositol 3 kinase (PI3K)/AKT and MAPK pathways is a well-recognized trait in cancer, heightened suppression of these signaling routes represents an attractive approach to strengthen regorafenib properties. Administration of AKT inhibitors (such as perifosine and MK2206) or PI3K inhibitors (such as PX-866) have individually been shown to cooperate with regorafenib to inhibit HepG2, Hep3B, and Huh7 liver cancer cells proliferation^32^.

Annexin A3 (ANXA3) is known to play a crucial role in promoting tumor aggressiveness, impeding apoptosis and inducing pro-survival autophagy in sorafenib-resistant HCC cells. Tong et al.^33^ have shown in sorafenib non-responsive HCC in vivo models that co-administration of regorafenib and an anti-ANXA-3 monoclonal antibody can enhance apoptotic induction because of the abolishment of autophagy. Similarly, navitoclax, a specific inhibitor of the Bcl-2 and Bcl-xL anti-apoptotic proteins, increased the sensitivity of Hep3B and HepG2 cells to regorafenib, as shown by increased general apoptotic features^34^.

Mitogens such as insulin-like growth factor 1 (IGF1) are involved in HCC growth. Simultaneous treatment with vitamin K1 (VK1), a non-toxic natural compound with anti-tumor properties, and IGF1 receptor (IGF1R) antagonists (such as GSK1838705A or OSI-906) enhanced the pro-apoptotic and anti-proliferative actions of regorafenib in vitro. This combination blocked the IGF1R downstream cascades MAPK and PI3K/AKT and significantly impaired cell migration through decreased actin polymerization^35^. Gankyrin, a protein that plays a crucial role in malignant cancer development, is responsible for desensitizing human HCC cells to chemotherapy by upregulating c-Myc and hence reprogramming glucose metabolism. Accordingly, the use of a glycolytic inhibitor (2-DG), a glutaminase 1 inhibitor (BPTES) or a c-Myc inhibitor (10058-F4), in addition to regorafenib, effectively inhibited the growth of HCC-PDX tumors with high gankyrin expression^36^.

Natural compounds, such as the above mentioned VK1, are promising resources for combined therapeutic strategies. Given its anti-tumor effects displayed in liver cancer, oleanolic acid coupled to regorafenib has been tested for advanced HCC. Both agents acted synergistically to inhibit the proliferation, EMT, migration and invasion of the PLC/PRF/5 cell line, which agrees with the suppression of tumor growth and lung metastasis in PLC-bearing mice^37^. In the same way, chlorogenic acid, a polyphenol present in the human diet, has shown positive effects in vitro in conjunction with regorafenib. Its co-administration caused beneficial effects on cell growth inhibition and apoptosis induction, as well as on cell cycle progression inhibition, MAPK and PI3K/AKT/mTOR signaling repression, and migrating cancer cell percentage decrease^38^.

On the other hand, the anti-cancer compound CDDP coupled to regorafenib synergistically inhibited HepG2 and Hep3B cells growth^28^. The combination of regorafenib plus metformin, the main drug used to treat type 2 diabetes, caused greater inhibition of MHCC97H HCC cells proliferation and stimulated cell death, also preventing tumor relapse and metastasis in an in vivo model. This two-drug treatment reduced the expression of hypoxia-inducible factor 2α (HIF-2α), a key factor in tumor cell adaption to oxygen depletion, which led to negative modulation of EMT by upregulation of the 30-kDa HIV Tat-interacting protein (TIP30)^39^.

All these preclinical studies support the synergistic activity of regorafenib in combination with different compounds (Table 1), resulting in enhanced anti-tumoral actions of this drug compared with the effects of single administration. These findings confirm the outstanding role of drug co-administration for improving regorafenib-based second-line treatment and thus the outcomes of late-stage HCC patients.

**Table 1:** Results of regorafenib-based combined treatments in preclinical HCC, CRC and GISTs models

Cancer type	Model	Regorafenib-combined treatment schedule	Global effects	Molecular effects	Reference
HCC	HepG2, Hep3B, and Huh7 cell lines	Perifosine, MK2206 or PX-866	Enhanced cell death	–	32
	Sorafenib-resistant HepG2 xenografts	Anti-ANXA3 monoclonal antibody	Tumor growth suppression Apoptosis induction Reduced autophagosome formation Inhibition of pro-survival autophagy	↓ ANXA3 and LC3-II	33
	Genetically engineered immune-competent sorafenib non-responsive HCC mouse model				
	HepG2 and Hep3B cell lines	Navitoclax (ABT-263)	Promotion of mitochondrial caspase-cell death	↓ Mcl-1 ↑ Bim, caspase 3 activity, cytochrome *c* release, PARP cleavage	34
	PLC/PRF/5, HLF and HepG2 cell lines	VK1 plus GSK1838705A or OSI-906	Enhanced anti-proliferative and pro-apoptotic effects of regorafenib Cell migration impairment via actin depolymerization	↓ AFP secretion (PLC/PRF/5 and HepG2) ↑ caspase 3/7 activation Loss of cytoplasm F-actin fibers but redistribution around de nucleus (HLF) ↓ p-ERK, p-AKT, p-p38, p-JNK, p-TSC2, p-S6 (PLC/PRF/5)	35
	HCC-PDX with high gankyrin levels	2-DG, BPTES or 10058-F4	Tumor growth repression	–	36
	PLC/PRF/5 cells	OA	Inhibition of cell growth, migration and invasion	–	37
	PLC-bearing mice		Reduction of tumor volume, lung metastasis, EMT, migration and invasion	↑ E-cadherin ↓ Vimentin, MMP-2, MMP-9	
	PLC/PRF/5 and HepG2 cell lines	CGA	Decreased cell proliferation and cell cycle progression from S to G2/M phase Apoptosis promotion Cell migration inhibition	↓ Ki-67 ↑ Annexin V, Bax, caspase 3/7 activation ↓ Bcl-2, Bcl-xL ↓ p-JNK, p-p38, p-S6, p-TSC2, p-ERK, p-AKT	38
	HepG2 and Hep3B cells	CDDP	Synergistical inhibition of cell growth	–	28
	MHCC97H cells	Metformin	Reduction of cell proliferation EMT suppression Apoptosis induction	↓ HIF-2α, N-cadherin ↑ TIP30, E-cadherin	39
	Orthotopic MHCC97H mouse model		Inhibition of postoperative recurrence and lung metastasis Apoptosis induction	↓ Ki-67, N-cadherin ↑ TUNEL positive cells ↑ TIP30, E-cadherin	
CRC	COLO205, HT29, LoVo, HCT15 cells	Pimasertib	Synergistic effects on growth inhibition	–	61
	HCT15 cells		Apoptosis induction	↓ p-MAPK, p-AKT, p-4E-BP1, p-p70S6K, cyclin D1 ↑ p27 ↑ cleaved caspase 3, PARP	
	SW620, SW480, HT29, and HCT116 cell lines	PX-866	Enhanced cell death	–	32
	HCT116 cells	MK2206			
	HCT116 mouse model	MK2206	Suppression of tumor growth	–	
	HCT116, SW480, HT29, and HCT116 p53^−/−^ cells	5-FU	Reduced cell viability	↓ Mcl-1, Bcl-xL ↑ PUMA (HCT116 p53^−/−^)	48
	5-FU resistant HCT116 (HCT116R) and DLD-1 (DLD-1R) cells	5-FU	Overcoming of 5-FU resistance Decrease of cell viability and tumor spheres formation	–	58
	DLD-1R mouse model		Inhibition of tumor growth and tumor spheres formation	↓ ABCG2, β-catenin, WNT1 ↑ Bax	
	Oxaliplatin-refractory CRC-PDX	Irinotecan	Tumor growth delay	–	50
	HCT116 cells	5-FU, oxaliplatin or cetuximab	Increased percentage of apoptotic cells	↑ PUMA	56
	Mice with HCT116 xenograft tumors	5-FU	Tumor volume decrease and apoptosis activation	↑ TUNEL positive cells, active caspase 3	
	HT29, SW620, LoVo, HCT15, SW48, SW480, HCT116, GEO and cetuximab-resistant GEO (GEO-CR), and SW48 (SW48-CR) cells	Cetuximab	Enhanced growth inhibition and apoptotic cells percentage (HT29, SW480, SW620, HCT116, LoVo, HCT15, SW48-CR, GEO-CR)	↓ p-AKT, p-S6, p-MAPK (SW480, SW620, SW48-CR, GEO-CR, HCT116, LoVo, HCT15)	51
	Subcutaneous HCT15, HCT116, GEO-CR, and SW48-CR xenograft mouse models		Greater tumor volume reduction	–	
	Orthotopic HCT116 xenograft mouse model		Inhibition of tumor growth in the cecum and metastasis formation Suppression of neovascularization	–	
	SW620, HCT116, and HT29 cell lines	FTD	Inhibition of FTD incorporation into DNA	↓ p-ERK ↓ TS	62
		FTD → regorafenib	Higher survival inhibition Lower FTD incorporation into DNA Apoptosis induction (SW620)	↑ cleaved PARP (SW620) ↓ p-ERK, TS	
	SW620 and COLO205 xenograft mouse models	FTD/TPI → regorafenib	Higher inhibition of tumor growth	–	
	HCT116, HCT116 p53^−/−^, RKO and HT29 cells	CRT0066101	Cell growth inhibition Clonogenic growth inhibition (HCT116 and RKO) Apoptosis induction (RKO)	↑ cleaved PARP (RKO) ↓ p-HSP27 (RKO) ↓ p-PKD2, p-AKT, p-ERK (RKO) ↓ p-PKD2 (HCT116) ↓ NF-κB activity (HCT116 and RKO)	63
	Mitoxantrone-resistant BCRP-overexpressing S1-M1-80 cells	Mitoxantrone or SN-38	Reversion of BCRP-mediated MDR Improvement of cells sensitivity to mitoxantrone or SN-38 Raised [^3^H]-mitoxantrone cellular retention via BCRP efflux impairment	Interaction with the BCRP transmembrane domain	64
	Mitoxantrone-resistant BCRP-overexpressing S1-M1-80 xenografts	Topotecan	Reduced tumor volume and weight	–	
	Doxorubicin-resistant ABCB1-overexpressing SW620 cells (SW620/Ad300)	Paclitaxel	Overcoming of ABCB1-mediated MDR Increased [^3^H]-paclitaxel cellular accumulation via ABCB1 efflux impairment	↓ ABCB1 ATPase activity Interaction with the ABCB1 transmembrane domain	65
	SW620/Ad300 xenograft mouse model		Synergistic effect on tumor growth inhibition Higher intratumoral paclitaxel concentration Increased plasma regorafenib concentration	–	
	SW620/Ad300 cells	Paclitaxel, doxorubicin or vincristine	Overcoming of ABCB1-mediated MDR Reduced resistance fold		
	HCT116 and SW620 cell lines	Lapatinib	Decreased survival rate Cell cycle arrest in G0/G1 phase (↑ G0/G1 phase cells and ↓ G2/M phase cells) (HCT116) Apoptosis induction	↓ cyclins A, B, D1, E, CDK1, CDK6 ↓ p-AKT, p-ERK, Bcl-2, Mcl-1, XIAP, survivin ↑ cleaved PARP, Bax	52
	Subcutaneous HCT116 xenograft mouse model		Tumor growth inhibition Diminished tumor volume and weight Inhibition of cell growth and angiogenesis	↓ Ki-67, p-AKT, CD34 ↑ cleaved caspase 3, Bax	
	HCT116 and HT29 human cells CT26 and MCC38 mouse cells	Sildenafil and neratinib	Elevated cell death Increased toxic autophagosome formation (HCT116 and CT26) Activation of death receptor signaling (HCT116 and CT26) Lysosomal disfunction and release of cathepsin B (HCT116 and CT26) Mitochondrial disfunction and release of AIF (HCT116 and CT26) Modulation of tumor cells immunogenicity via autophagy-dependent regulation of HDAC proteins (CT26 and MCC38)	↑ p-eIF2α, p-ATM, p-AMPK, p-ULK-1, p-S317, p-ATG13 (HCT116 and CT26) ↓ p-mTOR, p-AKT, p-p70S6K, p-ERK (HCT116 and CT26) ↑ ATG5, Beclin 1 (HCT116 and CT26) ↓ Mcl-1, Bcl-xL (HCT116 and CT26) ↓ p-GSK3, β-catenin (HCT116 and CT26) ↑ Frizzled (HCT116 and CT26) ↑ CD95 plasma membrane levels (CT26) ↓ HDAC proteins (CT26) ↓ PD-L1, IDO-1 (CT26 and MCC38) ↓ PD-L2 (CT26) ↑ MHCA (CT26 and MCC38)	66
	Mouse CT26 tumors		Enhanced reduction of tumor growth	–	
	D5D-knocking down HCA-7 colony 29 and HT29 cells	DGLA	Improvement of regorafenib inhibitory effect on cell viability and colony formation	–	67
	SW620, SW480, HCT15, HCT116, LoVo, SW48, GEO, SW48-CR, and GEO-CR	Silybin	Further cell growth suppression	–	68
	HCT15, SW480, SW48 and SW48-CR		Reduced colony formation Higher ROS generation Apoptosis induction	↑ cleaved PARP ↑ caspase 3, pro-caspase 9 (SW48, SW48-CR and HCT15) ↓ p-AKT, p70S6K, p-4E-BP1	
	EpCAM-positive HCT8 xenograft mouse model	CAR-modified NK-92 cells with specificity against EpCAM	Lower tumor volume and weight Increased persistence of NK cells in the tumor	–	69
GISTs	Imatinib-resistant GIST430-654 cells	TL32711 or LCL161	Increased pro-apoptotic activity	↓ p-KIT, p-AKT, cIAP1, XIAP, survivin ↑ cleaved PARP	78

*4E-BP1* eukaryotic initiation factor 4E binding protein 1, *5-FU* 5-fluorouracil, *ABCB1* multidrug resistance protein 1, *ABCG2 (BCRP)* ATP-binding cassette sub-family G member 2, *AFP* alpha-fetoprotein, *AIF* apoptosis inducing factor, *AKT* protein kinase B, *AMPK* AMP‐dependent protein kinase, *ANXA3* Annexin A3, *ATG5* autophagy related protein 5, *ATG13* autophagy related protein 13, *Bax* Bcl-2 associated X, *Bcl-xL* Bcl-2-like protein 1, *Bim* Bcl-2-like protein 11, *CAR* chimeric antigen receptor, *CD34* hematopoietic progenitor cell antigen CD34, *CD95* FAS cell surface death receptor, *CDK1* cyclin-dependent kinase 1, *CDK6* cyclin-dependent kinase 6, *CGA* chlorogenic acid, *cIAP1* cellular inhibitor of apoptosis protein 1, *CRC* colorectal cancer, *D5D* delta-5-desaturase, *DGLA* dihomo-γ-linolenic acid, *eIF2α* eukaryotic translation initiation factor 2α, *EMT* epithelial-to-mesenchymal transition, *EpCAM* epithelial cell adhesion molecule, *ERK* extracellular signal-regulated kinase, *FTD* trifluridine, *GISTs* gastrointestinal stromal tumors, *GSK3* glycogen synthase kinase 3, *HCC* hepatocellular carcinoma, *HDAC* histone deacetylase, *HIF-2α* hypoxia-inducible factor 2α, *HSP27* heat shock protein beta-1, *IDO-1* indoleamine‐pyrrole 2,3‐dioxygenase, *JNK* c-Jun N-terminal kinase, *Ki-67* proliferation marker protein Ki-67, *LC3-II* microtubule-associated protein 1 light chain 3 II, *MAPK* mitogen-activated protein kinase, *Mcl-1* induced myeloid leukemia cell differentiation protein, *MDR* multidrug resistance, *MHCA* major histocompatibility complex A, *MMP-2* matrix metalloproteinase-2, *MMP-9* matrix metalloproteinase-9, *mTOR* mammalian target of rapamycin, *NF-ĸB* nuclear factor-ĸB, *NK* natural killer, *OA* oleanolic acid, *p* phospho, *p38* p38 MAPK, *p70S6K* ribosomal protein S6 kinase, *PARP* poly(ADP-ribose) polymerase, *PD-L1* programmed cell death-1 ligand 1, *PD-L2* programmed cell death-1 ligand 2, *PDX* patient-derived xenograft, *PKD2* protein kinase D2, *PUMA* p53-upregulated modulator of apoptosis, *ROS* reactive oxygen species, *S6* S6 ribosomal protein, *TIP30* 30 kDa HIV Tat-interacting protein, *TPI* tipiracil, *TS* thymidylate synthase, *TSC2* tuberin, *TUNEL* terminal deoxynucleotidyl transferase dUTP nick end labeling, *ULK-1* unc-51 like autophagy activating kinase 1, *VK1* vitamin K1, *WNT1* Wnt family member 1, *XIAP* X-linked inhibitor of apoptosis

## Regorafenib and CRC

### Therapeutic approaches for advanced CRC

Globally, CRC is the second cancer with higher mortality rate and the third in terms of incidence, which means that ~1 of every 10 cancer cases and deaths is due to CRC^13^. One of the main risk factors is age, with a positive correlation between the increasing aging population and CRC incidence^40^. Inadequate lifestyle habits, such as excessive alcohol consumption, smoking, high intake of red processed meat^13,40,41^, obesity, diabetes^41^, and inflammatory bowel disease also contribute to the development of this cancer^40,41^. In addition, CRC has a hereditary component in which Lynch and familial adenomatous polyposis syndromes are included^40^. Metastasis exists in 25% of patients at the time of diagnosis, and it is estimated that 50% of tumors metastasize in the short term^4,9,42^. Although advanced-stage CRC patient survival has more than doubled in the last 20 years^43^, the 5-year survival of these individuals is ~10%^41^. Many therapies have been recently developed; however, 5-fluorouracil (5-FU) is still the mainstay of advanced CRC treatment^42^.

Single 5-FU intravenous administration has been the only available therapy for advanced CRC over several decades, but basic first-line treatment options currently comprise combined regimens involving 5-FU plus cytotoxic agents such as oxaliplatin and irinotecan. The combination of these compounds has resulted in the chemotherapy regimens FOLFOX (5-FU + leucovorin + oxaliplatin), FOLFIRI (5-FU + leucovorin + irinotecan), and FOLFOXIRI (5-FU + leucovorin + oxaliplatin + irinotecan)^40,42^. There is also the possibility of replacing 5-FU by oral-administered capecitabine in the FOLFOX and FOLFIRI therapies, thus generating CAPOX (or XELOX) and CAPIRI regimens, respectively^11^. All of these therapeutic strategies have yielded great tumor growth management^40^ and increased the median progression-free survival (PFS) to 8 months^9^. The PFS increased to 9–12 months with posterior incorporation into the pre-existing first-line regimens of molecularly targeted drugs against VEGFR and epidermal growth factor receptor (EGFR)^9^. Using one or other targeted biologic inhibitor depends on the molecular profile of the tumor^4^. While the anti-VEGFR monoclonal antibody bevacizumab is used for patients with RAS mutant advanced CRC, cetuximab and panitumumab, two anti-EGFR monoclonal antibodies, are approved for individuals with RAS wild-type disease^11,44^ (Fig. 1).

Upon tumor progression, chemotherapy regimens not employed as first-line treatment should be given in the second-line setting^11^. Ramucirumab, an anti-VEGFR2 monoclonal antibody, or aflibercept, a recombinant fusion protein targeting placental growth factor, can also be introduced into the combined protocols to enhance treatment efficacy. Despite the improvement in survival rates, patients with advanced CRC inevitably develop chemoresistance^11,42^. If patients in this situation maintain good organ function and performance status, they are candidates for regorafenib or trifluridine/tipiracil (FTD/TPI or TAS-102) administration^4,42,44^. Moreover, the FDA recently granted accelerated approval to pembrolizumab, and nivolumab alone or in combination with ipilimumab (an anti-cytotoxic T-lymphocyte antigen-4 inhibitor), in the second- and later-setting for patients with microsatellite instability-high (MSI-H) or deficient DNA mismatch repair metastatic CRC that has progressed following typical chemotherapy^44^ (Fig. 1).

Regorafenib as monotherapy is the standard treatment for refractory advanced CRC^11,40^. The international, multicenter, randomized, placebo-controlled phase 3 CORRECT trial showed for the first time the benefit of regorafenib in patients with metastatic CRC that progresses after all available therapies. Regorafenib treatment significantly increased the median OS to 6.4 months from 5.0 months in the placebo group (HR = 0.77, CI 0.64–0.94, *p* = 0.0052)^45^, implying that this drug can reduce death risk by 23%^4^. Given these positive outcomes, the FDA approved regorafenib in 2012 to become the first drug applicable in patients after conventional therapy failure^4,9,44^. Regorafenib efficacy and safety were also evaluated in a randomized, double-blind, placebo-controlled, phase 3 trial (CONCUR) performed in Asian patients with refractory metastatic CRC. This study reported an OS of 8.8 months in the treated group versus 6.3 months in the placebo group (HR = 0.55, CI 0.40–0.77, *p* = 0.00016)^4,9,46^. The difference between the OS values in both clinical trials can be explained by the fact that patients enrolled in the CORRECT trial received more pre-treatment and were further deteriorated than those in the CONCUR study^4,42^.

Although there is a large list of approved agents for treating advanced CRC, searching for more potent and effective treatments will continue to prolong patient survival beyond 30 months^4,11,42–44^.

### Regorafenib for advanced CRC: evidences in preclinical models

A study performed with the human CRC cell lines HCT15, DLD-1, HT29, and HCT116 showed a drastic decrease in cell viability when regorafenib was administered at doses higher than 5 µM^47^, an effect that was also observed in SW480^48^, KM12SM, Caco-2, LoVo, WiDr, and RKO CRC cells^49^. Cell proliferation inhibition was also corroborated in a panel of in vitro models^50–52^, as well as in an HCT116 nanoimprinting 3D culture^53^ and in cell lines established from CRC primary tumors^54^. Moreover, daily regorafenib administration at 30 mg/kg suppressed tumor growth in a highly aggressive murine CT26 metastatic colon cancer model^55^. Although similar results were observed in different HCT116-bearing mouse models^53,56^, it has been noted that a lower regorafenib dose, such as 10 mg/kg/day, can delay tumor growth^47^, even in oxaliplatin-refractory CRC-PDX^50^.

Regarding apoptosis status after regorafenib treatment, several researches have shown that this drug induces apoptosis both in vitro^48,57,58^ and in vivo^55,59^. In this way, regorafenib has been shown to downregulate phospho-STAT3 and its pro-survival targets cyclin D1 and Mcl-1 and upregulate cleavage of PARP and caspase 9 in different CRC cells. Regorafenib-mediated STAT3 inhibition was due to an increase in SHP-1 tyrosine phosphatase activity by direct impairment of the association between N-SH2 and the catalytic domain of SHP-1, as we have already described in the HCC section. These findings were reported in not only CRC cells but also in HCT116 xenograft models^47^. Another investigation using 5-FU-resistant in vitro models supports the regorafenib-mediated downregulation of the oncogenic STAT3 pathway, finding that it takes place in parallel with the inhibition of its upstream molecule phospho-mTOR^58^.

It is known that p53-upregulated modulator of apoptosis (PUMA) evokes CRC cell apoptosis. Chen et al.^56^ reported that regorafenib treatment enhances PUMA expression in HCT116, Lim2405, LoVo, Lim1215, SW48, and RKI CRC cells in a dose- and time-dependent manner and in xenograft tumors; moreover, this effect correlated with apoptosis induction. Further analyses revealed that activation of PUMA-mediated apoptosis occurs via ERK inhibition^56^, the subsequent activation of glycogen synthase kinase 3β (GSK3β) and finally the binding of the p65 NF-ĸB subunit to the proximal PUMA promoter^56^. According to these findings, regorafenib administration also causes ERK downregulation in different in vitro^50^ and in vivo models^49^.

FGFR signaling participates in cancer cell proliferation, angiogenesis, and migration. Cha et al.^57^ observed that regorafenib treatment of NCI-H716 CRC cells leads to the inhibition of FGFR2 phosphorylation and its downstream molecules, abrogating pro-survival FGFR2 signaling. With respect to tumor vascularization and angiogenesis inhibition, regorafenib significantly decreased the microvessel area and VEGFR2- and TIE2-positive vessels in a highly aggressive murine metastatic CRC model^55^. Similarly, this drug suppressed tumor vascularity and tumor perfusion in HT29 carcinoma xenografts^59^, in a KM12SM-bearing mouse model^49^ and in CRC-PDX models^50^.

Regorafenib at clinically effective concentrations exhibits a potent inhibitory effect on the migration ability of CRC cells^49^, thus decreasing pro-EMT vimentin levels^58^ and reducing small tumors spread in the abdominal cavity in vivo^53^. There is evidence that SHP-1 impedes transforming growth factor β1 (TGF-β1)-induced EMT through phospho-STAT3 downregulation. Because regorafenib activates SHP-1, which results in low phospho-STAT3 levels, it is not surprising that the drug shows anti-EMT activity via targeting this pathway, inhibiting invasion in vitro and lung metastasis in vivo^60^. In agreement, regorafenib has also been shown to avoid liver metastasis^50,55^ and other extra-hepatic nodule formation in vivo^50^, in addition to decreasing the infiltration of tumor-associated macrophages, which are crucial for angiogenesis and metastatic spreading^55^. Moreover, when bone marrow-derived mesenchymal stem cells (MSCs) migrate to the tumor stroma, they differentiate into carcinoma-associated fibroblasts (CAFs), stimulating cell proliferation and invasion. A study using KM12SM + MSCs-bearing mice revealed that regorafenib completely blocks lymph node metastasis, inhibits tumor growth and stromal reaction, and prompts apoptosis^49^.

On the other hand, experiments employing CRC cell lines resistant to 5-FU reported a meaningful miR-34a-associated reduction in colon cancer stem-like phenotypes after regorafenib addition. This effect was related to decreased tumor sphere formation and side populations and reduced expression of the stemness markers Wnt family member 1 (WNT1) and Notch receptor 1 (Notch1)^58^.

Numerous preclinical researches have demonstrated the broad activity of regorafenib in advanced CRC. Similar to the results reported in advanced HCC, regorafenib mainly abrogates tumor cell proliferation and promotes apoptosis, in addition to reducing tumor vascularity and invasion ability (Fig. 2). This evidence suggests that regorafenib is a useful therapeutic option for refractory metastatic CRC.

### Emerging combined treatment strategies with regorafenib against advanced CRC

Some studies have analyzed the potential effect of regorafenib co-treatment with other compounds. This could provide new therapeutic insights that could reinforce the anti-tumoral potency of regorafenib to prolong CRC patient survival. The combination of regorafenib with the selective MEK1/2 inhibitor pimasertib synergistically reduced the proliferation of pimasertib-resistant CRC cells and induced apoptosis, which were associated with the inhibition of the pro-survival intracellular signals MAPK and AKT^61^. Similarly, the AKT inhibitor MK2206 and the PI3K inhibitor PX-866 have separately been demonstrated to augment regorafenib lethality in colon cancer cells^32^.

The co-administration of regorafenib with other drugs approved for CRC has been evaluated. Regorafenib coupled with 5-FU caused a greater decrease in HCT116, HT29, and SW480 cells growth^48^. In 5-FU-resistant in vitro and in vivo models, this combination also inhibited the appearance of cancer-starting cell phenotypes through Wnt/β-catenin signaling impairment, which thereby reversed 5-FU resistance^58^. Moreover, regorafenib combined with irinotecan showed greater tumor growth inhibition of oxaliplatin-refractory CRC-PDX^50^. Regorafenib was also able to increase cell sensitivity to oxaliplatin and cetuximab in vitro and to 5-FU in vivo through the induction of PUMA-mediated apoptosis^56^. Similarly, a study using cetuximab-resistant CRC cell lines reported that regorafenib overcomes primary and acquired resistance to anti-EGFR therapy by targeting the MAPK and AKT pathways^51^. The chemosensitizer ability of regorafenib against anti-EGFR inhibitors was also supported by an assay using GEO and SW48 cetuximab-resistant xenograft mouse models^51^. The combination of both drugs has also been demonstrated to be effective in HCT116 orthotopic xenografts, as shown by growth regression and neovascularization and metastasis abolishment^51^. In addition, the combination of regorafenib plus FTD revealed that cell survival is reduced to a higher extent when FTD is administered prior to regorafenib; this can be explained by a greater decrease in phospho-ERK1/2 and thymidylate synthase (TS) expression, which induces cell death^62^. These findings are supported by increased cytotoxic effects in SW620 and COLO205-bearing mice when FTD/TPI is added followed by regorafenib^62^.

Protein kinase D (PKD) constitutes a key mediator of multiple biological processes implicated in cancer. It has been found that co-treatment with a PKD-specific inhibitor (CRT0066101) exerts synergistic effects against CRC cells through the inhibition of proliferation and clonogenic growth and the activation of apoptosis. These results were associated with the suppression of PKD2-mediated RAS/RAF/ERK, PI3K/AKT and NF-ĸB signaling cascades^63^. Breast cancer resistance protein (BCRP or ABCG2) participates in the acquisition of resistance to multiple drugs (MDR). Zhang and colleagues^64^ observed that regorafenib improves BCRP-overexpressing S1-M1-80 CRC cells sensitivity to mitoxantrone and SN-38, both substrates of BCRP, due to its interaction with the BCRP transmembrane domain and its ability to impair BCRP efflux and increase intracellular drug retention. Regorafenib and topotecan also increased cytotoxicity against BCRP-overexpressing CRC xenografts^64^. Likewise, regorafenib overcame MDR in in vitro and in vivo CRC models that overexpress multidrug resistance protein 1 (ABCB1)^65^. Wang et al.^65^ confirmed that regorafenib stimulates cell sensitivity to paclitaxel, an ABCB1 substrate, increasing the intracellular paclitaxel level via inhibition of ABCB1-associated chemotherapy efflux.

Lapatinib, a TKI approved for breast cancers that overexpress HER2 (or ERBB2), has been shown to improve anti-tumor regorafenib properties against metastatic CRC. Combined treatment meaningfully arrested the cell cycle in the G0/G1 phase and decreased the expression of several cyclins and cyclin-dependent kinases (CDKs) in vitro. This treatment also modulated the apoptotic balance leading to cell death and suppressed tumor growth and angiogenesis in an HCT116 xenograft tumor model^52^. Another research focused on triple treatment with regorafenib plus sildenafil (a phosphodiesterase 5 inhibitor) and neratinib (an ERBB1/2/4 inhibitor) found a more than additive inhibitory effect on CRC cells growth in vitro and in vivo, enhancing harmful autophagosome formation, death receptor signaling activation and mitochondrial and lysosomal dysfunction. Furthermore, these agents increased the levels of major histocompatibility complex A (MHCA) and reduced the expression of biomarkers related to tumor immune response, such as PD-L1^66^.

With respect to the co-administration of regorafenib plus natural substances, a study employing dihomo-γ-linolenic acid (DGLA) has shown this molecule to complement the anti-cancer properties of regorafenib in CRC cells with knockdown of delta-5-desaturase, the enzyme that converts DGLA into arachidonic acid^67^. Regorafenib was also tested in conjunction with silybin, a natural plant extract that has biological activity, in several CRC cell lines, such as HCT15, SW480 and cetuximab-resistant and cetuximab-sensitive SW48 CRC cells; this combination was found to increase the inhibition of cell growth and colony formation and promote apoptosis. These results were associated with the inactivation of PI3K/AKT/mTOR signaling and the increased production of reactive oxygen species (ROS)^68^.

Finally, regarding immunomodulatory activity, which could allow the reactivation of the immune response against cancer cells, Zhang et al.^69^ verified that co-administration of regorafenib with chimeric antigen receptor (CAR)-modified NK-92 cells (CAR-NK-92) with specificity against epithelial cell adhesion molecule (EpCAM) displays beneficial anti-cancer properties in EpCAM-positive HCT8 tumor xenografts.

Simultaneous administration of regorafenib with other drugs or natural compounds has been shown to enhance the anti-cancer properties of regorafenib alone by targeting some of the major tumor-related pathways or increasing the intracellular drug concentration (Table 1). Despite the significant results of regorafenib monotherapy, a combination strategy represents a breakthrough in therapeutic approaches for advanced stages of CRC.

## Regorafenib and GISTs

### Therapeutic approaches for advanced GISTs

GISTs are unusual neoplasms, accounting for 0.1–3% of all gastrointestinal malignant tumors^12,70^, and they represent the most common mesenchymal cancer in the digestive tract^1,12,70–74^. GISTs annual incidence is approximately 14–20 per million in Northern Europe^72^ and 10–15 per million in the United States^1,12^, with nearly 5000 new cases diagnosed each year^3,74^. The most frequent locations where these tumors originate are the stomach (60%) and small intestine (30%)^2,3,12^. Although GISTs can appear at any time, advanced age is considered the principal risk factor, as most GISTs arise after 60 years of age^70,72^. Nearly 15–50% of patients display advanced and metastatic disease^12,70^, for which there was formerly no effective treatment due to poor response to classical cytotoxic drugs^1,74^.

Imatinib constitutes the first-line treatment for metastatic and unresectable GISTs^70–73,75^, being able to raise advanced GISTs patients OS to ~60 months versus 18 months achieved with traditional therapies^3,70,72,73^. For those individuals who do not tolerate imatinib or present primary or secondary imatinib resistance, sunitinib is available in the second-line setting^1,2,12,73,75^. Unfortunately, almost all metastatic GISTs ultimately also develop sunitinib resistance. For these reasons, regorafenib received FDA approval in 2013 as a third-line treatment, becoming the only functional therapy for advanced GISTs when both imatinib and sunitinib fail^1,3,12,71,73,75^ (Fig. 1). Regorafenib approval was based on positive outcomes derived from an international, multicenter, prospective, randomized, placebo-controlled phase 3 trial (GRID) enrolling patients with GISTs refractory to both imatinib and sunitinib. This trial showed an increased PFS (4.8 months in the regorafenib arm versus 0.9 in the placebo arm, HR = 0.27, CI 0.19–0.39, *p* < 0.0001) and improved disease control rate (52.6% for regorafenib versus 9.1% for placebo) in the regorafenib group^76^. Currently, several studies testing new treatment options for advanced GISTs, either immunotherapy or novel molecular targeted agents, are underway^1,71^.

### Regorafenib in single or combined treatment for advanced GISTs: evidences in preclinical models

Few studies evaluating regorafenib effects on in vitro and in vivo GISTs models have been performed (Fig. 2). Van Looy et al.^77^ reported that regorafenib suppresses tumor growth in a GIST-PDX model (UZLX-GIST9) through necrosis induction, microvessel density decrease and KIT, AKT and the eukaryotic initiation factor 4E binding protein 1 (4E-BP1) inactivation. Experiments conducted with imatinib-resistant GIST430-654 and GIST48 cells support the pro-apoptotic activity of regorafenib, as evidenced by KIT and AKT inhibition, survivin and XIAP downregulation and cleaved PARP upregulation. In addition, the combination of regorafenib plus TL32711 or LCL161, both mimetics of second mitochondria-derived activator of caspases (SMAC), displayed agonistic anti-cancer properties against GIST430-654 cells^78^.

Few investigations have been performed with regorafenib alone or co-treatment for advanced GISTs. Co-administration of regorafenib with SMAC mimetics has exhibited beneficial cytotoxic effects against this cancer (Table 1). Nonetheless, further studies are required to determine the mechanisms underlying the actions of regorafenib in GISTs as a third-line therapy and, overall, to evaluate possible regorafenib-based combinations with the objective of enhancing the last treatment option for this tumor type.

## Conclusions and future perspectives

Regorafenib represents the gold standard for advanced HCC, CRC, and GISTs when first-line or even second-line therapies fail. This article summarizes the broad subset of anti-tumor actions exhibited by regorafenib in preclinical models of three cancer types, highlighting its anti-proliferative, pro-apoptotic, anti-angiogenic, anti-metastatic and immunomodulatory properties, as well as its ability to regulate autophagy and stemness markers (Fig. 2). Regorafenib effectiveness is clear and well-established given the preclinical outcomes discussed here and the clinical evidence that resulted in regorafenib approval. Nevertheless, because regorafenib is employed in the final stages when tumors become refractory to standard chemotherapy, it would be advantageous to search for new treatment strategies that enhance OS beyond regorafenib monotherapy. Among these strategies, co-administration of this drug with other agents capable of potentiating its efficacy by targeting the same signaling pathways or even complementary cancer-related routes has arisen as a promising therapeutic approach. In this review, we describe favorable results obtained from the investigations that have been carried out to date that evaluate combined regorafenib-based treatments (Table 1).

In regard to improving regorafenib via combination therapies, it is important to note that some of the principal causes underlying loss of chemotherapy sensitivity and resistance acquisition are augmented ABC transporter activity^65^, the hypoxic microenvironment^18^, immune response evasion^31^, and miRNA expression modulation^79^.

ABC transporter proteins are responsible for the acceleration of drug efflux from tumor cells. Thus, the exploration of ABC transporters mediating regorafenib efflux will be critical for the design of new combined therapies able to increase its intracellular concentration, leading to the considerable enhancement of its anti-cancer actions. Likewise, due to the important role played by the hypoxia response in chemotherapy resistance, especially in HCC patients where hypoxia appears as a direct consequence of the anti-angiogenic activity of sorafenib, it would be interesting to co-administer regorafenib with compounds targeting the oxygen-deficient microenvironment. For example, co-treatment with regorafenib plus hypoxia-activated pro-drugs (HAPs), agents that facilitate oxygen delivery in tumor hypoxic areas (e.g., YQ23), or compounds targeting the main mediators of the cell response to hypoxia, the hypoxia-inducible factors (HIFs)^18^.

Immunotherapy for cancer-specific antigens has emerged as one of the main interests in the cancer therapy landscape. In particular, immune checkpoint inhibitors such as nivolumab and pembrolizumab, both human antibodies targeting PD-1, have become useful for treating advanced HCC and CRC after first-line treatment failure. Accordingly, future studies focused on the combination of regorafenib with nivolumab, pembrolizumab or novel drugs that impede immune response escape by cancer cells represent a great approach to optimizing the effects of regorafenib through immunomodulation enhancement^31^.

Another promising research direction aimed at improving anti-tumor regorafenib properties could be the administration of this drug in conjunction with antagonists or mimetics of well-known cancer-related miRNAs. The first case proposed refers to inhibitors targeting miRNAs with oncogenic actions, whereas the mimetics option is based on the functional re-establishment of miRNAs whose tumor-suppressor role has been lost^79^.

On the other hand, because cancer cells are prone to acquire drug resistance and because regorafenib seems to be effective after previously unsuccessfully administered chemotherapy, several in vitro and in vivo studies have analyzed the effects of regorafenib on tumors for which it is not yet indicated. Encouraging outcomes have been observed in gastric cancer^57,80,81^, adenoid cystic carcinoma^82^, bladder carcinoma^83^, breast cancer^84^, thyroid, prostate and endometrial neoplasms^85^, lung squamous cell carcinoma^86^, meningioma^87^, multiple myeloma^88^, and neuroblastoma^89^.

Although further preclinical studies will assist in providing novel insights into the subjacent molecular mechanisms of regorafenib activity as well as the suitability of new combination strategies, the transfer of the basic and translational findings detailed in this article to the clinical area is also an important need. This will undoubtedly enhance the therapeutic potency of regorafenib and extend its employment to other cancers where providing new treatment options is of utmost importance.
